# Hospital Reports

**Published:** 1887-06

**Authors:** A. C. Broell


### Hospital Reports.

### MERCY HOSPITAL, CHICAGO.

### CASE OF BONE GRAFTING IN THE LOWER JAW.

### SERVICE OF JOHN S. MARSHALL, M.D., DENTAL AND ORAL SURGEON.

(Reported by A. C. Broell, M. D., Interne.)

Miss S., aged forty-two years, presented herself for operation on January 26, at the Mercy Hospital. Her previous history is about as follows: Nine years ago she was suffering with a disease of the lower jaw, which was diagnosticated osteo sarcoma, by a homœopathic physician, who afterwards performed an exsection for the removal of the disease. The piece exsected extended from the first bicuspid tooth backwards to, and included one-half an inch of, the ramus. This physician also made an attempt to bring the fragments of the jaw into apposition, so that bony union might occur; no such union, however, resulted, but the jaw was so displaced that the median line of the chin was situated about one-half an inch to the right of the median line of the face.

Later on the patient began to suffer with neuralgic pains about the left temporo-maxillary articulation, and in the right arm. To relieve this pain, which was thought to be dependent upon displacement of the jaw, an operation was performed by Dr. Marshall some time ago, by means of which the cicatricial tissue was removed through the mouth, and reposition of the jaw was accomplished. The fragments were held in position by a gold rod screwed into the ramus, and attached to a gold cap applied to the first bicuspid tooth, and retained by oxyphosphate cement.

The patient this day (January 26) presented herself for the second operation, by means of which the gap now existing between the fragments, one inch and a half in extent, it was hoped, might be replaced with new tissue reproduced by bonegrafts. Having been previously prepared for operation, she was at once etherized. An incision was made about four inches in length in the line of the old scar, down to the mucous membrane lining the oral cavity. Both ends of the fragments were laid bare, and well scraped. The hæmorrhage which followed, though slight, was persistent, but was finally checked with a hot solution of bichloride of mercury (1:1000 of water).

At the same time, having etherized a half grown rabbit, both its femurs were laid bare, and with bone cutting forceps twelve small pieces of bone, ranging in size from about two to six lines in length, two to three lines in width, and one line in thickness, having the periosteum attached, were removed from the epiphyseal extremities. These pieces were placed in a solution of the bichoride of mercury (1:1000), which was kept at a temperature of 99° to 100° F., where they remained from five to ten minutes. After all oozing had stopped these pieces were transferred from the solution to the wound, being placed in two rows in such a way that the inner row had the periosteum towards the mucous membrane and the outer one had it towards the cutaneous surface, the ends of the rows being in contact with the denuded ends of the jaw-bone, and the cancellated structure of the pieces being in contact.

The incision was now closed with eight sutures of carbolized silk, with a few twisted strands of silk left in the lower end of the wound for drainage. The wound was dressed with iodoform and antiseptic gauze covered with oiled paper, the whole dressing being held in place by a bandage. Throughout the operation antiseptic precautions were scrupulously carried out.

The patient left the hospital on the following day. At home she improved rapidly, the wound healing by first intention throughout four-fifths of its extent; in the other fifth, near the drainage, the stitches cut through, retarding the healing somewhat. The remaining stitches and the silk left for drainage were removed on the sixth day. The entire wound healed without the formation of pus.

As far as can be ascertained at present, there are fair prospects that the operation will prove successful.—*Medical Progress.*

